# The forensic implications of food hypersensitivity – a review of cases in United Kingdom courts: January 2014–February 2020

**DOI:** 10.1177/17579139221136723

**Published:** 2022-12-02

**Authors:** MH Gowland, MJ Walker

**Affiliations:** Allergy Action, St Albans, UK; Visiting Fellow, School of Medicine, University of Southampton, Southampton SO16 6YD, UK; Laboratory of the Government Chemist, Teddington, UK; Institute for Global Food Security, School of Biological Sciences, Queen’s University Belfast, Belfast, UK

**Keywords:** food hypersensitivity, food allergy, coeliac, prosecution, regulation, food law, inquest, coroner, food labelling, food safety, offence, health and safety, manslaughter

## Abstract

**Aims:**

Food allergy is a major public health concern. Failures of food allergen avoidance and the consequences for those with food hypersensitivity (allergies, intolerances and coeliac disease) have a forensic context. The aim of this study was to collate and analyse the use of action in the United Kingdom (UK) courts as redress following adverse food allergy reactions or failures of allergen management.

**Methods:**

Details of prosecutions during the study period (1 January 2014 to 31 January 2020) were recorded from regular key word Internet searches. National and local news reports were primary sources, along with commentary from enforcement and regulatory professionals. Information was also collected from coroners’ inquests by attending hearings and direct contact with coroners and participants in the hearings. Freedom of Information requests were made to local authority enforcement departments. In several cases, the authors had direct involvement in investigations.

**Results:**

From 2014 to 2020, there was an increase in reports. Seventy prosecutions were recorded as well as two associated appeals and two applications for Hygiene Emergency Prohibition Notice. This resulted in 68 convictions; seven individuals received custodial sentences, three of which were suspended although one individual had a tagged curfew imposed. Fines ranged from £50 to £93,000. Details of the law applied and the evidence gathering processes are reported.

**Conclusion:**

Legal action, including landmark prosecutions for Gross Negligence Manslaughter and Preventing Future Deaths reports from coroners, with salience of criminal penalties, has led to changes in labelling law and improved allergen management practices better to protect the interests of patients with food hypersensitivities. A central system of collation of such data, and on ‘near misses’, will enable more focused root cause analysis to further improve allergen management and reduce patient risk.

## Introduction

Food allergy is a major public health concern. Food hypersensitivity avoidance (allergies, intolerances and coeliac disease) has a legislative (Table 1) and forensic context. Contravention of legislation may lead to prosecution and conviction of food business operators.

**Table 1:** 

Legislative context for allergen management	
Jurisdiction and statute	Provisions as they apply to allergens^a^
**European legislation** ^b^	
Regulation (EC) No 178/2002 . . . *on the general principles and requirements of food law* . . .^1^ Implemented domestically by The Food Safety and Hygiene Regulations 2013.	Food businesses must provide safe food ‘determining whether any food is injurious to health’ should take into account the ‘particular health sensitivities of a specific category of Consumers’.
Regulation (EU) No 1169/2011 *on the provision of food information to consumers* . . . (The ‘Food Information (to Consumers) Regulations’ (FIR)) Implemented domestically by the Food Information Regulations 2014.	Identifies 14 priority allergens (Annex II) (‘Substances or Products Causing Allergies or Intolerances’) determined at EU level from a global list of eight priority allergens set by Codex Alimentarius. Only two have quantified limits; sulphur dioxide (sulphites) >10 mg/kg (as SO_2_) and gluten-free foods may be so labelled so if their gluten content is below 20 mg/kg. FIR requires prepacked foods to bear a legible ingredients list in regulated text size, with any priority allergens present highlighted and the information made available throughout the supply chain from business to business and to the final consumer via labelling, other written information or dialogue with staff supported by signage. Food businesses operators are required to keep, manage and make available correct and up to date accurate, consistent and verifiable information about which allergens had been included in which product or dish. From 1 October 2021 a UK-specific amendment to FIR requires previously exempt items prepared and packed on site (prepacked for direct sale, PPDS) to carry the product name and full ingredients with any of the 14 priority allergens highlighted.
Regulation (EU) No 852/2004 *on the hygiene of foodstuffs*	Requires food business operators to identify hazards in their activities and put in place procedures to control them
**UK (only) legislation**	
The Food Safety Act 1990, (Chapter 16)	Provides for criminal offences including rendering food injurious to health (Section 7), selling, to the purchaser’s prejudice, food which is not of the nature or substance or quality demanded (Section 14) and falsely or misleadingly describing or presenting food (Section 15)
The Health and Safety at Work Act 1974 (1974 c 37) Section 3	Requires businesses to ensure that people not employed by them, but who may be affected by their business activities are not exposed to risks to their health or safety, and also to provide such people with information about their business activities to ensure their health and safety.

EU: European Union.

a For authoritative text see EUR-Lex, https://eur-lex.europa.eu/homepage.html.

b On UK Exit day (11 pm on 31 January 2020) extant EU law was transposed into UK legislation.

In the UK consequences of non-compliance with the law described in Table 1, include criminal sanctions, a Food Information Regulations Improvement Notice (FIRIN), a proven breach of which is a criminal offence. A temporary Hygiene Emergency Prohibition Notice (HEPN) may be used if there is an imminent food hypersensitivity risk to health.^1,2^ A coroner investigating a food allergy fatality may issue a ‘Regulation 28 Preventing Future Deaths’ (PFD) report^3,4^ to named food businesses and other bodies for response, and openly published.^5^

To date, there is no regulation specifically governing Precautionary Allergen Labelling (PAL) (‘May contain’) where allergens not used as ingredients may be present as contaminants.^6^ However, the general risk of allergen cross-contamination is recognised in hazard analysis and risk assessment in food businesses.

Eight sample food allergy cases from UK courts were published in 2014.^7^ These included an initial conviction for supplying peanut labelled as almond. The business later successfully appealed the conviction.

This study includes UK court reports from January 2014 to the end of January 2020. Its purpose is to understand the circumstances in which prosecutions may (and may not) take place, the law used, the food allergens involved, the evidence and the penalties applied. In addition, background data may inform those at risk and their advisers to improve understanding and reduce risks.

## Method

Data were collected from 1 January 2014 for continuity with our previous work^7^ to 31 January 2020, when the COVID-19 pandemic supervened, by regular Internet searches using key words (Supplementary Material Table S1) and national and local news reports, and recorded (Supplementary Material Table S2) with occasional commentary from professional (regulatory) organisations and other stakeholders. Full details of the two cases involving Gross Negligence Manslaughter are available on public websites.^8,9^ Information was collected from coroners’ inquests by attending in person and through direct contact with coroners, families, clinicians, local and national regulators and others (MHG). MHG has also been directly involved in some investigations. Charges were confirmed for some cases through 19 Freedom of Information requests to local authorities. One new source of dissemination is social media, cases reported online sometimes in real time via Twitter™ and elsewhere.

## Results

**Figure 1:**
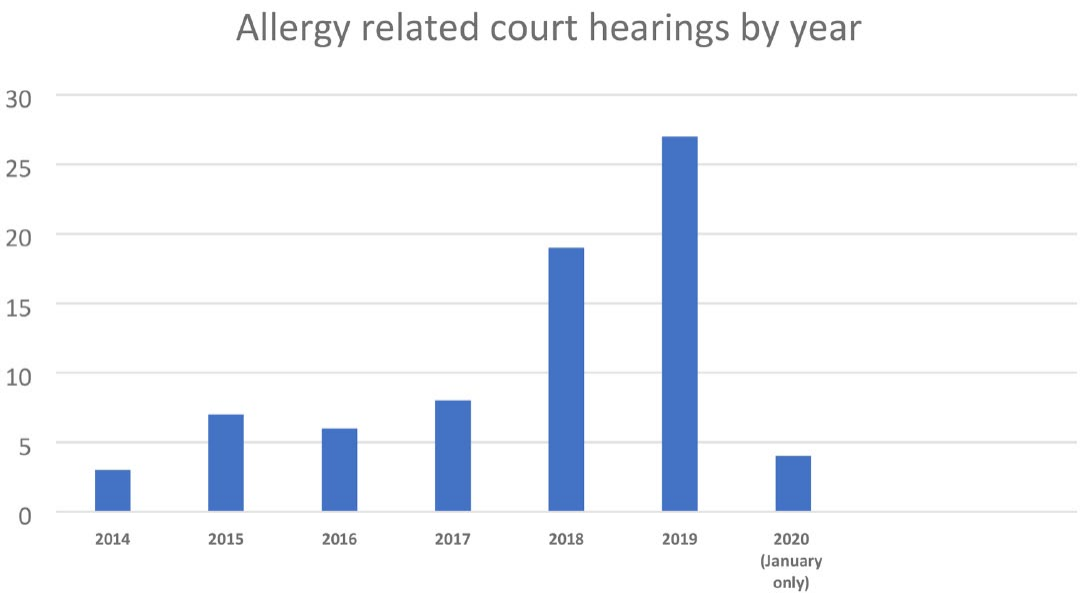
Allergy-related UK court hearings from 1 January 2014 to 31 January 2020

There has been a steady increase in the number of food allergy court hearings since 2014 (Figure 1). Food allergy-related prosecutions (*n* = 70), two associated appeals and two applications for HEPN were recorded in the United Kingdom from 1 January 2014 to 31 January 2020.

One catering outlet (a ‘takeaway’) was acquitted of food allergy-related offences because undeclared peanut was due to the fault of their supplier (which ceased trading). Of the 69 remaining cases, 4 involved prepacked and labelled foods, 64 were takeaway or restaurant dishes and 1 was prepacked for direct sale (PPDS). In all, 68 cases led to convictions. In four reports, the takeaway order was made using a delivery platform.

In two cases, prepacked imported food was not labelled in English. One prepacked product had no allergen labelling, and one was a product containing unlabelled milk, which caused a severe reaction in a young child with milk allergy.

The UK geographical spread of the 68 convictions is shown in Table 2.

**Table 2:** 

UK geographical spread of allergen-related criminal convictions from 1 January 2014 to 31 January 2020			
Nation	Number of convictions		
England	51		
		Of which	
		North West of England	14
		North East of England	11
		Midlands	12
		South East of England	12
		South West of England	2
Wales	14		
Northern Ireland	2		
Scotland	1		
Total	68		51

## Which Food Allergens were Involved?

**Figure 2:**
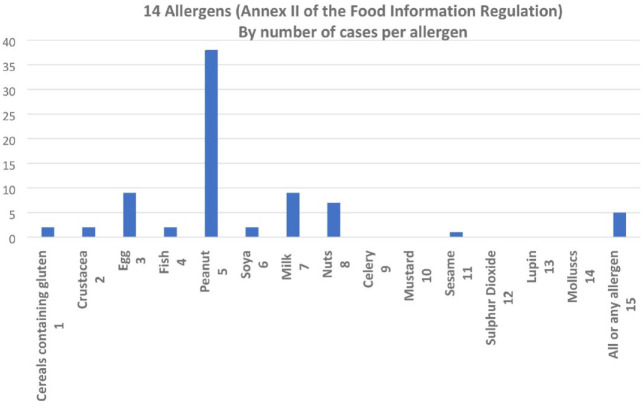
Number of cases by priority allergen

Five prosecutions involved failure to label, provide information or signage generally for all food allergens. The most common allergen cited in prosecutions was peanut, followed by egg and milk. All cases cited allergens in the Annex II list (Figure 2).

## Which Courts were Involved?

Most cases were heard by a bench of lay magistrates. Four were before a District Judge. Sixteen cases were heard in the Crown Court, owing to the gravity of the charges, or because the defendant availed of the right for the case to be so heard. Two cases proceeded to the Court of Appeal.

## Who Took the Prosecutions?

The investigation of three recorded allergen fatal reactions involved the police; in North Yorkshire following the death of Paul Wilson^8^ and in Lancashire following the death of Megan Lee,^9^ which both led to prosecutions for Gross Negligence Manslaughter, and in Bath following the death of Chloe Gilbert.^10^

The remaining prosecutions were taken by local authority trading standards officers (TSOs), and/or environmental health officers (EHOs). Some businesses, and particularly those with multiple sites, may have a primary authority arrangement with one local authority to access assured guidance and tailored advice on meeting regulations. In general, this can bring laudable consistency and efficiency to the regulatory process. However, on occasion there is tension between a prosecuting authority and a primary authority.^11^

## Which Law was Used?

**Figure 3:**
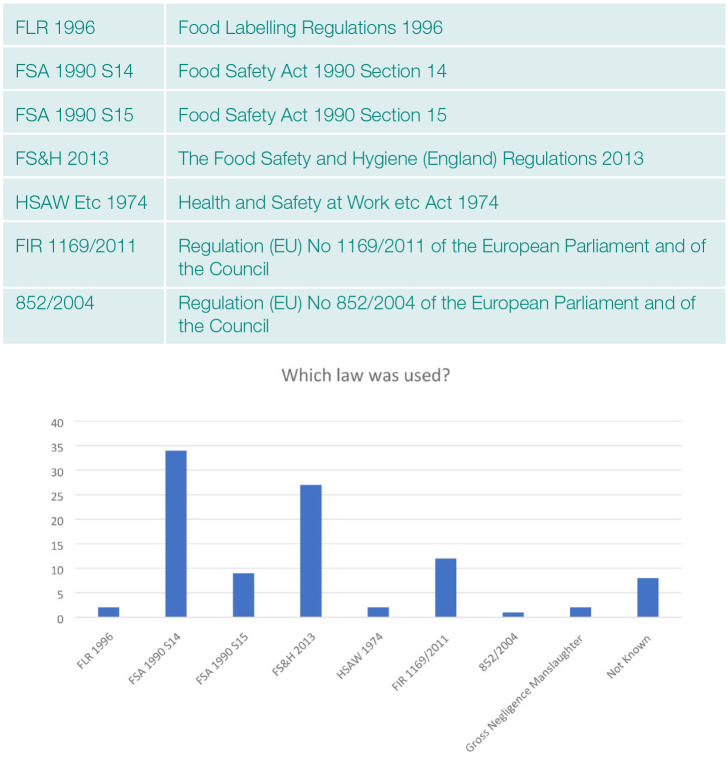
Which law was used?

The applicable law in the recorded prosecutions varied; most involved alleged offences under more than one regulation (Figure 3). One case specifically mentioned Regulation (EU) No 852/2004, which relates to the management of food safety, two cases involved Gross Negligence Manslaughter. In eight cases, details of the legal measures cited in relation to the alleged offences were not recorded in the news reports, or local authority press releases and could not be elicited by direct contact with those involved (Figure 3).

## Evidence – Test Purchases

Evidence included data obtained through analysis of a test purchase or food samples (*n* = 41) collected front and back of house during an investigation, food labels, menus, signage, product specifications and promotional information in physical and electronic format, including emails, websites and from telephone, Internet or digital platform ordering. In some cases following a reaction, if the food was no longer available, the business was asked to re-create the meal ordered under observation to be analysed for the suspected allergen. Lip swabs and other postmortem food samples were analysed. Dialogue between investigating officers, food business operators and other staff provided key background evidence about allergen policies and procedures. Photographs of signage and other information, kitchen, storage, service and delivery practices were also used. Businesses which had been advised not to sell food to people with a particular allergy were telephoned to test whether they would implement this instruction. Although rarely reported, food analysis was assumed generally to have been carried out in official food control (Public Analyst)^12^ laboratories.

## Convictions

Only two acquittals were recorded, though it remains challenging to gather acquittal information which, being of less media interest, is infrequently reported and has no public protection benefit in alerting consumers to potential risks. From anecdotal evidence, some local authority investigations of allergen incidents may not have proceeded to prosecution owing to disparity in legal support between the authority and the intended defendant.

## The Sentencing Guidelines

The Sentencing Council for England and Wales^13^ developed Sentencing Guidelines which came into force on 1 February 2016 in England and Wales. In Northern Ireland, Sentencing Guidelines for Food Safety and Hygiene offences were also published.^14^ (The Scottish Sentencing Council was established in October 2015 but has not to date addressed Health and Safety or Food Safety offences.)

The Sentencing Guidelines take into account the seriousness of the crime committed and the harm caused, the culpability, the risk posed by the offending behaviour, the way in which the individual has interacted with the local authority prior to the offence, the financial circumstances of the defendant (as an individual) and (through its directors) the business turnover and number of employees.

## Penalties

**Custodial Sentences:** Four individuals in three cases received custodial sentences. Three individuals in two cases received suspended prison sentences and one also had a tagged curfew between 9 pm and 7 am for 3 months.
**Community sentences:** One individual was required to do 300-h community service over 12 months. Two individuals in another case were required to do 100 h each over 12 months and another individual, 150 h over 12 months. Two individuals with suspended sentences were also required to do 120 h each. Another individual was required to do 20 days over 12 months.
**Fines:** Where data were available, fines ranged from £50 for a street trader who failed to make allergen information available to £93,000 for a large manufacturer supplying a product with undeclared egg. Of the 62 cases where fines were reported, the average was £6189, and the average costs awarded were £2063. Thirty-eight cases mentioned a Victim Surcharge.^15^ During the study period, these ranged from £20 to £170 with an average of £97.

## PFD Reports

PFD reports following allergy-related deaths include the report for Jackie Scott^16^ who was allergic to peanut and died in 2013 after eating a curry suspected to contain peanut. The published report does not include a reply from the business which supplied the food. More recently, a report was published following the death of Owen Carey after he ate chicken containing milk (having requested food without milk).^17^ This report was addressed to the restaurant chain involved and others.

In autumn 2018, following the widely publicised inquest into the death of Natasha Ednan-Laperouse, the PFD Report^18^ addressed alleged inadequate labelling of PPDS foods. This led to a statutory measure requiring PPDS foods to carry full ingredients and allergen labelling.^5^ This law (informally known as *Natasha’s Law*) is in force from October 2021.

## Prosecution Abroad

The death of a UK citizen on holiday in Italy in 2015 from hypoxic brain injury and cardiac arrest after consuming milk or a milk derivative was recorded in a UK inquest during which it was reported that a prosecution in an Italian court led to a suspended sentence for a waitress found guilty of manslaughter.^19^

## Discussion

Food-hypersensitive consumers should be able to access correct information about all relevant allergen ingredients in their food, including those which are not listed in Annex II of FIR. Anaphylaxis Campaign (AC) data indicate that kiwi, banana, legumes such as peas, beans, lentils and chickpeas are more commonly avoided by its AC members than some of those listed on Annex II – for example mustard, celery, lupin, molluscs (Personal communication to MHG from Anaphylaxis Campaign helpline manager, 11 July 2017). In addition, such consumers need to be able to discuss and assess the controls in place to prevent allergen cross-contamination throughout the supply chain.

The prosecutions for Gross Negligence Manslaughter were significant and involved inadvertent consumption of peanut in Indian-style cuisine. Close partnerships between the police and local authority food officers and prompt and thorough evidence collection led to custodial sentences. Peanut allergy is common and reports of deaths and ‘near misses’ to unexpected peanut in curries date back to 1988.^20,21^

The Health and Safety at Work Act 1974 (S.3) does not impose a time limit for laying information. This is helpful (e.g. in the Lancashire case) because fatal (and ‘near miss’) reaction investigations are complex, often taking more than the year allowed to bring charges under food law.

The Food Safety Act (S.14) offence has been widely used and generally depends on proof (e.g. from analysis or sometimes from ingredients information) that the allergen was present, following a request for their absence. The Food Safety and Hygiene Regulations,^2^ ‘Placing unsafe food on the market’, can be used even where a food sample is not available for analysis.

Accessing case reports often depends on news journalists who report from local Magistrates and District Judge hearings but may not always record full details of the charges or penalties. In 2017, it was possible to download Local Authority and Food Standards Agency (FSA) prosecution outcomes from the FSA website including details of the businesses, offences, penalties and local authorities, and to search for key words of likely relevance to the protection of food-hypersensitive consumers. This information no longer seems to be accessible, a shortcoming in research and trend analysis on local authority legal activity generally and food hypersensitivity prosecutions in particular.

The advent of the Sentencing Guidelines led to a requirement to demonstrate not just actual but potential harm requiring Public Analysts and other experts to explain to magistrates, juries and judges the likely or possible impact of the unlabelled presence of a food allergen.

The study period covers major reductions in national and local food control budgets^22^ although, encouragingly, local authorities still respond to consumer complaints and undertake sampling and analysis^23,24^ to identify unlabelled allergens. However, it has sometimes taken a severe or even fatal reaction to ensure that funding for such initiatives is available. Similarly, local authorities continue to engage with businesses, helping them to undertake allergen risk assessment and management, delivering formal and informal training. Many also engage with one another through regional workshops to ensure that they are up to date on food hypersensitivity issues and best practice, and some have reviewed the Memorandum of Understanding in place between Environmental Health and Trading Standards Officers involved in food control to improve investigation of complaints and support businesses (Personal communications MHG and Helen Dodds, Food Safety Manager, Hyndburn Borough Council. From 18 July 2018 and on-going).

In general, defendants were food business operators, but it is interesting to note one prosecution of a staff member under the Food Safety Act who sold a pizza containing milk (with vegetarian cheese) for a child with a declared milk protein allergy when he had promised ‘vegan’ ‘cheese’ without milk. He is reported to have pressed the wrong button on the ordering terminal. The child suffered a reaction requiring hospitalisation and the staff member tried (unsuccessfully) to change the order retrospectively on the terminal. The food business was able to demonstrate responsibility lay with the staff member rather than the business. This case serves as a salutary lesson for catering businesses and for staff training.

The current trend for plant-based or ‘vegan’ products represents a risk to those consumers who are highly sensitive to milk and eggs, but who may not wish to ‘make a fuss’. Businesses providing plant-based food may not be able to eliminate these key allergens at parts per million levels required for the most sensitive allergic consumers. While ingredients may not include egg or milk, without the kind of ‘positive release’ and batch testing controls used in the production of ‘free from’ foods, the necessary segregation is unlikely to be in place.

Food business reputations are increasingly shaped online. Companies with online ordering and delivery platforms now require businesses to implement higher food safety standards to appear on their sites^25^ which in turn is causing businesses dependent on their platform-presence to undertake training and more effective food safety risk assessments. Some platforms now require customers declaring a food hypersensitivity to contact the business directly, and not purchase via their platform. Some restaurants and takeaways will not provide food for delivery because although they can implement controls within their businesses, they cannot guarantee its allergen integrity while in the care of the delivery driver.

Finally, individuals who are undergoing or who have recently suffered an allergic reaction attributable to food may well report their reaction in real time on Facebook™, Twitter™, Instagram™, Trip Advisor™ or elsewhere. Public complaints and allegations suggesting that a business may be responsible for a reaction may lead to reputational damage which could have a significant impact on their trade, and lead to further intervention by local authority food officers.

## Conclusion

From 2014 to 2020 in the UK, there has been a steady increase in local authority investigations leading to prosecution and conviction of food business operators for offences involving unidentified food allergen presence, and failure to retain, manage, advertise and provide correct allergen information. Takeaway businesses selling unidentified peanut in curry represent a real risk to consumers and are still the focus of sampling projects in many areas of the UK. Similarly, unidentified egg in Chinese cuisine has led to prosecutions following sampling programmes in Wales. Reports of only two (manufactured product) prosecutions in Northern Ireland and one case of peanut in curry in Scotland during the study period may indicate less allergen enforcement activity, or use of alternative approaches to controlling allergen risks in local businesses.

The FIR are being used in enforcement and cases have been taken for failure to make information available or failure to have a notice inviting consumers to ask about allergens. The Food Safety and Hygiene Regulations^2^ provide a clear offence of selling unsafe food which has been used where the allergen information provided is incorrect.

Key investigations of takeaway businesses in the north of England have led to landmark prosecutions for Gross Negligence Manslaughter and related food offences, while major restaurant brands have received requests for PFD reports from coroners following high-profile inquests in London and elsewhere.

Purchasing and selling behaviour is changing. The use of digital platforms to order food for delivery ready to eat or for later consumption has required a major review of risks throughout the supply chain, and significant effort to ensure brand protection continues. High-profile cases, the apparent increase in young adults with multiple food allergies (particularly milk, egg and sesame), media interest and the immediacy of social media all play their part. More recently, ordering and delivery platforms have excluded food-hypersensitive customers from their services, insisting instead they attend the business and order in person. This is intended to reduce the risk of misunderstanding the request, or tampering with the order during delivery. However, as ordering online is now a way of life for many, particularly during the COVID-19 pandemic, there is a risk that some consumers will order without declaring their allergen avoidance need, preventing optimal allergen controls being implemented.

## Recommendations

The data reported here have been collected primarily through public domain sources which limit the extent of data analysis. In autumn 2021, the FSA and Food Standards Scotland (FSS) announced a pilot scheme to collect and investigate reports of food hypersensitive reactions,^26^ including where possible analysis of food samples. Data on the foods, amounts of allergen, communication, preparation and service practices involved may be gathered and with further engagement with local authority teams, regulatory advisory and enforcement action may lead to improved root cause analysis, lessons learned and consumer, business and wider public awareness to reduce risk. Pilot ‘Citizen Science’ projects have been commissioned by the FSA and UK Research and Innovation (UKRI)^27^ to engage food-hypersensitive communities to improve food safety standards in online food procurement. The FSA is also supporting research to establish the UK Anaphylaxis Registry and to continue to monitor anaphylaxis trends in the UK and beyond.^28^ Finally, the FSA through the British Society for Allergy and Clinical Immunology (BSACI) is funding the UK Fatal Anaphylaxis Registry (UKFAR) to review fatal anaphylaxis cases, optimising understanding and potentially reducing risks.^29^

Future best practice in food allergen management and the investigations of its failure would benefit from an overarching meta-analysis of the outcomes of the above studies augmented by official collection, collation, review and reporting of court cases and research on the disparity between legal resources available to local authorities to mount complex food allergen mismanagement prosecutions. Otherwise, systems opportunities for root cause analysis and associated lessons learned may be missed and inadequate allergen management practices unchallenged.

Food businesses, catering organisations and hospital trusts may also collect instances of food allergy reactions, including ‘near miss’ occurrences. Existing Health & Safety legislation has established the ‘Reporting of Injuries, Diseases and Dangerous Occurrences Regulations’ (RIDDOR), to report certain serious workplace accidents, occupational diseases and specified dangerous occurrences (near misses)^30^ that ought to include food allergy incidents, while General Practitioners (GPs) must report cases of food poisoning to their local environmental health department. Systematic collation of health-related ‘near miss’ data collected and investigation of the datasets generated are key to optimising root cause analysis for food allergy adverse incidents.
